# Spectroscopic Analysis of the TiO_2_ Nanoparticles Influence on the Interaction of 5,10,15,20-(Tetra-4-carboxyphenyl)porphyrin with Human Serum Albumin

**DOI:** 10.3390/ijms27010554

**Published:** 2026-01-05

**Authors:** Andra Dinache, Ana Maria Udrea, Mihai Boni, Adriana Smarandache, Angela Staicu

**Affiliations:** Laser Department, National Institute for Laser, Plasma, and Radiation Physics, 409 Atomistilor Str., 077125 Magurele, Romania; andra.dinache@inflpr.ro (A.D.); ana.udrea@inflpr.ro (A.M.U.); mihai.boni@inflpr.ro (M.B.)

**Keywords:** photodynamic therapy, photosensitizer, porphyrin, TCPP, TiO_2_ nanoparticles, binding constant, human serum albumin, molecular docking

## Abstract

Photodynamic therapy is a cancer treatment that relies on a photosensitizer (PS) to generate reactive oxygen species upon light activation, thereby destroying cancer cells. The photophysical properties of porphyrins make them effective PSs, while nanoparticles (NPs) enhance their delivery and stability. The bioavailability and targeting efficiency of NPs-PS complexes may be improved through transport via human serum albumin (HSA). This study investigates the HSA binding affinity with 5,10,15,20-(Tetra-4-carboxyphenyl)porphyrin (TCPP) and with TiO_2_-TCPP complexes. The interactions were analyzed using UV-Vis absorption, laser-induced fluorescence (LIF), and FTIR spectroscopy. Molecular docking was performed and provided consistent binding constant values for the TCPP–HSA complex with UV-Vis absorption measurements. LIF data revealed a slightly lower affinity when compare free porphyrin with TiO_2_-TCPP, possibly due to competitive binding between TiO_2_ and HSA. Docking simulations indicated that TCPP favorably interacts with amino acid residues located in subdomains IB and IIIA of HSA, supporting a preferential binding near Sudlow site I. FTIR measurements revealed conformational changes in HSA for both its interactions with TCPP and TiO_2_-TCPP, including alterations in α-helical content and reorganization of the hydrogen bonding network within the polypeptide backbone.

## 1. Introduction

Photodynamic therapy (PDT) is a promising cancer treatment due to its minimal invasive nature, targeted application, and limited adverse effects [1,2,3]. PDT relies on photosensitizers (PSs) that generate reactive oxygen species upon light activation, leading to selective cancer cell destruction [3,4].

Among the diverse PSs suitable for PDT, porphyrins stand out due to their photophysical properties [1,5]. Some porphyrin derivatives, based on tetrapyrrole structures with added functional radicals, exhibit high singlet oxygen quantum yields and strong absorption at longer wavelengths, making them ideal for cancer treatment [6,7]. Still, one of the major drawbacks of porphyrin-based PSs is their low water solubility that results in a tendency to aggregate in aqueous environments, which diminishes their photoactive potential and can lead to skin photosensitivity [3,6,8,9]. To overcome these challenges, various strategies have been explored, including the introduction of covalent modifications such as PEG chains, carboxylate, and sulfonate groups, as well as the alteration of peripheral substituents and core structures. These modifications enhance aqueous solubility, reduce aggregation, improve stability and selectivity, and ultimately facilitate more efficient porphyrin delivery in biological environments [6]. Several strategies that imply micelles, liposomes, polymers, lipids as nanocarriers or metal oxides, ceramics, silica, metal–organic framework (MOF) nanoparticles, and carbon nanotubes were employed to develop nanoplatforms to enhance the PS delivery, reduce toxicity and increase treatment efficacy. Among these, due to their ability to improve drug stability and extend systemic circulation time, nanoparticles and liposomes are promising approaches in cancer therapy [3,10,11].

Nanoparticles (NPs), such as titanium dioxide (TiO_2_), have shown great promise in addressing these limitations. These nanostructures can accommodate surface modifications for improved PS binding, improve the solubility of hydrophobic PSs and enable targeted delivery to cancer cells [12,13].

Although TiO_2_ nanoparticles show potential for drug delivery, their clinical translation is limited by dose, size, and crystal phase-dependent cytotoxic, genotoxic, and immunogenic effects. These risks, together with evidence of systemic distribution, organ accumulation, and their IARC and WHMIS classification as potentially carcinogenic and hazardous, underscore the need for precise control of particle size, crystal phase, and surface chemistry. Surface functionalization strategies and rigorous long-term in vivo studies of biodistribution, clearance, and chronic toxicity are therefore critical to improving biocompatibility and defining safer conditions for future biomedical applications [14,15,16,17].

On the other hand, a critical factor in ensuring the successful delivery and bioavailability of PS–nanomaterial complexes is their interaction with biological molecules, particularly proteins. Among these, human serum albumin (HSA), the most abundant plasma protein, plays a pivotal role in drug transport and biodistribution. The PS interaction with HSA, including its affinity, binding site, and binding mode, controls its biodistribution and, ultimately, the PDT efficacy. These factors together determine the PSs’ capacity to be transported in the human bloodstream. Therefore, it is of specific interest to study the binding of HSA with any potential PS intended for PDT [18,19]. The structure of HSA has three homologous domains (I, II, and III), each one divided into two subdomains (A and B) with six and four α-helices, respectively [20]. According to Sudlow et al. [21], two main protein–drug binding pockets were found. Sudlow site I belongs to the subdomain IIA, while Sudlow site II is situated within subdomain IIIA. Differences in size, polarity, shape, and solubility all have an impact on interactions between PSs and HSA binding sites [22].

The photophysical properties of porphyrins are highly sensitive to the surrounding microenvironment. Interactions with HSA can significantly influence the optical and photochemical behavior of porphyrins. These effects are attributable to the hydrophobic nature of HSA’s primary binding sites (IIA and IIIA), as well as to the proximity of specific amino acid (AA) residues. Investigating these interactions is essential for optimizing the efficacy of PDT. Previous studies have characterized the interactions of various porphyrins with HSA using a combination of biophysical techniques, such as UV-Vis absorption, fluorescence, circular dichroism (CD), and Fourier-transform infrared (FTIR) spectroscopy [23,24,25]. Docking tools are also important to identify drug binding sites on HSA and assess binding strength [26].

Nanoparticles can modify a drug’s binding affinity to HSA, influencing its biodistribution and therapeutic performance, sometimes improving targeting and reducing toxicity. Although nanocarrier-based delivery systems have enhanced the efficacy of hydrophobic drugs, many have failed in clinical trials because nano–bio interactions have been overlooked. In the bloodstream, nanocarriers rapidly interact with biomolecules, especially proteins, forming a protein corona that alters their physicochemical properties and ultimately their biological behavior and efficacy [27].

In this study, we explore the interactions between HSA and 5,10,15,20-(Tetra-4-carboxyphenyl)porphyrin (TCPP), a porphyrin-based PS, in comparison with its complexes with TiO_2_ NPs. TCPP carries a negative charge due to its deprotonated carboxyl groups, and like other anionic porphyrins, it binds more strongly to serum albumins than cationic analogs. Albumins are valuable drug carriers because they are abundant in blood, bind diverse molecules, and naturally accumulate in tumors, improving photosensitizer delivery. However, albumin binding can also alter the photosensitizer’s excited-state behavior and ROS generation, influencing overall treatment efficacy depending on the binding site [28].

By employing spectroscopic techniques such as UV-Vis absorption, laser-induced fluorescence (LIF), and FTIR, alongside molecular docking simulations, we explore the binding behavior and photophysical changes associated with these interactions. Insights into the photophysical parameters of the protein–ligand complexes were obtained by studying the kinetics of the phosphorescence of generated singlet oxygen. Since ibuprofen is used as a model ligand for *in silico* studies of HSA binding, we additionally performed molecular docking simulations with ibuprofen to compare its binding site and interaction pattern with those of TCPP [29]. The comparison allows us to better contextualize the binding mode of TCPP in the context of a well-characterized HSA ligand. These insights contribute to understanding how TCPP and TiO_2_-TCPP complexes interact with HSA, underlying their binding, and they can be relevant for advancing in new approaches to cancer treatment.

## 2. Results and Discussion

### 2.1. Computational Results

**Figure 1:**
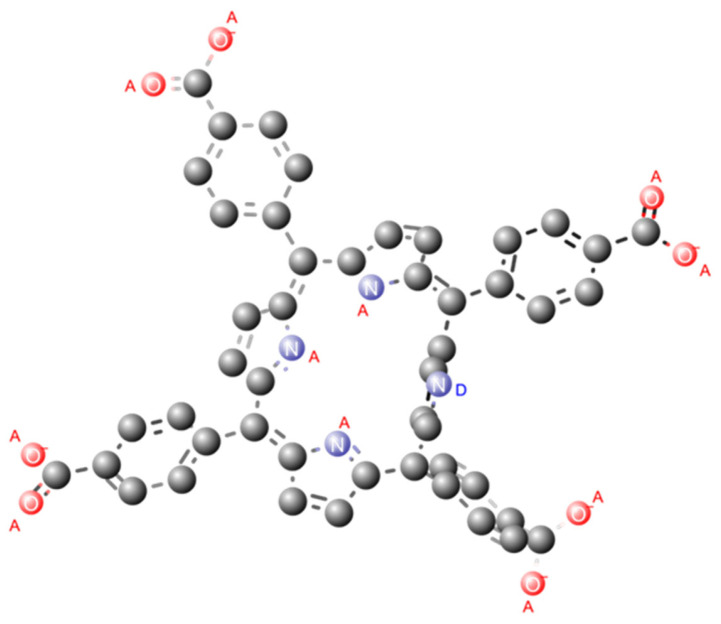
A 3D structure of TCPP with highlighted donor and acceptor atoms with A for acceptor atoms and D for donors. The structure was optimized and visualized using MarvinSketch software [30].

TCPP has eleven acceptor atoms, of which three are in the tetrapyrrole ring and eight in the carboxylic acid group. This structure also has one donor atom, located in the tetrapyrrole ring. The H-bond donor/acceptor count was calculated using Marvin Sketch 21.20 software [30] at a pH of 7.40 as shown in Figure 1.

#### Molecular Docking

A binding energy greater than −6 kcal/mol suggests no biological effect [31,32]. Blind molecular docking predictions show a low free energy of binding (−7.17 kcal/mol), indicating a good biological affinity between TCPP and HSA. According to predictions, the estimated inhibition constant (K_I_) between HSA and TCCP is 5590 nM, corresponding to a binding constant (*K_b_*) of 1.78 × 10^5^ M^−1^.

**Figure 2:**
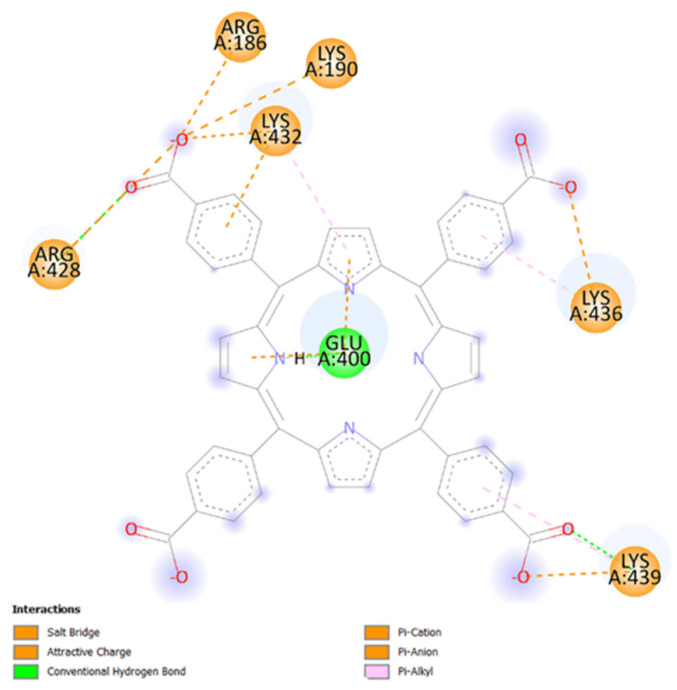
A 2D visualization of the blind molecular docking results that show the interactions between TCPP and HSA AAs residues. The image was obtained using Discovery Studio visualizer software, version V21.1.0.20298 [34].

As shown in Figure 2, TCPP and HSA interact favorably by forming Pi–cation, Pi–anion, salt-bridge, attractive charge, Pi–alkyl, and conventional hydrogen-bound interactions with AA residues from subdomains IB and IIIA. Table 1 synthesizes the type of interactions between TCPP and HSA AA residues, along with the corresponding distances between atoms.

Hydrogen bonds with donor–acceptor distances of up to 3.4 Å are typically considered strong [33]. Our findings show that TCPP forms conventional hydrogen bonds with the AAs GLU400, ARG428, and LYS439. Additionally, salt bridges and attractive charge interactions are observed with AAs residues ARG186, LYS190, ARG428, LYS432, LYS436, and LYS439. Pi–anion and Pi–cation interactions are established with GLU400 and LYS432, while hydrophobic Pi–alkyl interactions occur with LYS432, LYS436, and LYS439.

AA residues GLU400, ARG428, LYS432, LYS436, and LYS439 are located in the subdomain IIIA of HSA, close to Sudlow site II, whereas ARG186 and LYS190 belong to the subdomain IB.

Our docking results for the HSA–TCPP complex indicate that only some carboxyphenyl groups and also interactions with the nitrogen and carbon atoms from the porphyrin core form defined electrostatic and hydrogen–bond interactions with HSA. According to our simulations, one carboxyphenyl group appears accessible to the solvent.

The interaction model for TCPP on anatase TiO_2_ presented in Figure S1 also suggests that binding occurs through interactions with the carboxyphenyl groups or interactions with the nitrogen and carbon atoms from the porphyrin core. However, these simulations suggest that TCPP probably does not use all its potential anchoring sites in either complex, and may leave functional groups available for additional interactions in the TiO_2_–TCPP…HSA system.

**Figure 3:**
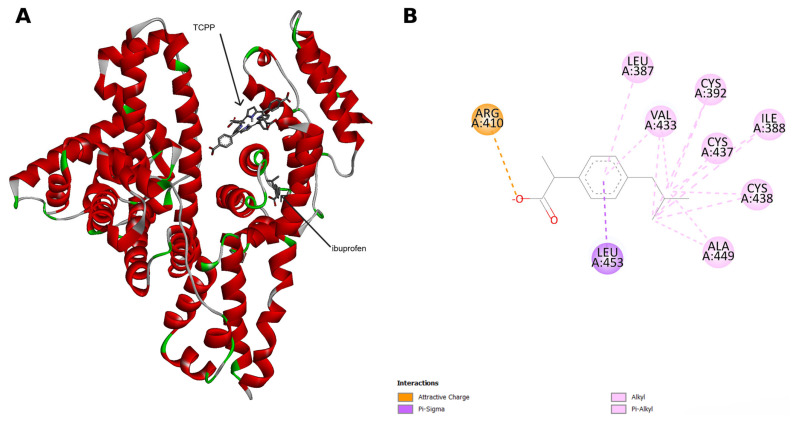
(**A**) Superposition of the TCPP–HSA and ibuprofen–HSA docking complexes. (**B**) Two-dimensional representation of the interactions between ibuprofen and HSA.

To compare TCPP with a classical HSA ligand, we also performed molecular docking simulations with ibuprofen. Comparative analysis indicates that TCPP and ibuprofen occupy different binding regions on HSA (Figure 3A).

According to Evoli et al., one of the preferred binding sites of ibuprofen is Sudlow’s site II [29]. Our docking predictions indicate that the ibuprofen pose is stabilized by one predominant electrostatic (attractive charge) interaction with ARG410, complemented by several alkyl and Pi–alkyl interactions with aa residues LEU387, ILE388, CYS392, VAL433, CYS437, CYS438, ALA449, and LEU453 (Figure 3B). The lowest free energy of binding −7.92 kcal/mol for ibuprofen, is more favorable than that obtained for TCPP (−7.17 kcal/mol). Overall, these results suggest that TCPP and ibuprofen bind HSA with comparable affinity but in distinct binding sites: TCPP in subdomains IB and IIIA near Sudlow site II, whereas ibuprofen in the classical Sudlow site II pocket.

Comparative analysis with the docking literature data indicates that the binding mode found for TCPP is consistent with previously reported porphyrin–HSA complexes. Chaves et al. [35] utilized molecular docking to predict the interaction between HSA and 10,15,20-Tetra(pyridine-4-yl)porphyrin (4-TPyP) and the HSA main binding sites. Their calculations revealed that the most favourable interactions occurred when 4-TPyP bound to Sudlow site I of HSA, forming interactions with residues LYS195, TRP214, ARG218, HIS288, GLU292, ASN295, LYS444, PRO447, and VAL455 [35]. In another study, Sułkowski et al. [33] also employed molecular docking to investigate the interactions between protoporphyrin IX (PpIX), a hydrophobic photosensitizer, and HSA, utilizing the same PDB structure of HSA (1AO6). Their findings indicate that PpIX binds in the tertiary structure of HSA in the subdomains IB and IIA and interacts with AA residues: ARG117, TYR138, ILE142, HIS146, PHE149, PHE157, TYR161, ARG186, and LYS190 [33]. Zunszain et al. present the crystal structure of HSA–hemin–myristate complex (PDB ID: 1o9x). The binding site of hemin pocket in subdomain IB is defined by interactions with AA residues: ARG114, ARG117, TYR138, HIS146, TYR161, and LYS190 [36]. Similar to these reports, we identified that TCPP binds to the AA residues ARG186 and LYS190.

Other report shows that the 10,15,20-tetrakis(2,6-difluoro-3-sulfophenyl)porphyrin (TDFPPS_4_) porphyrin binds to subdomain IIA (Sudlow site I) of HSA and forms favorable interactions with AA residues ALA191, LYS195, TRP214, ARG218, ASN295, ASP340, LYS444, and ASP451. This study revealed that HSA and TDFPPS4 binds superficially around TRP214 residue without altering the secondary structure of the protein. The porphyrin remains partially exposed to solvent and may undergo aggregation in the bloodstream [23]. Molecular modeling reported by An et al. [24] suggests that the meso-tetrakis(4-hydroxyphenyl)porphyrin (THPP) could be able to partially incorporate into Sudlow site II of subdomain IIIA of HSA by hydrophobic interactions and hydrogen bonding.

Strózik et al. investigated the interaction between 5,10,15,20-tetrakis(4-sulfonatophenyl)porphyrin (TPPS) and HSA and found that TPPS forms hydrogen bonds with TRP214, ASN295, and LYS436 and is additionally stabilized by salt bridges with LYS190, ARG218, LYS432 and LYS439 [37]. In our model, TCPP displays attractive charge and Pi–alkyl interactions with LYS436 and forms salt bridges with LYS190 and LYS439, closely mirroring the interaction pattern observed for TPPS. Taken together, these findings suggest that TCPP shares common binding features with other anionic porphyrins, engaging overlapping sets of residues in subdomains IB and IIIA of HSA.

### 2.2. Spectroscopic Analyses

#### 2.2.1. Determination of the Binding Affinity Through UV-Vis Absorption Spectroscopy

**Figure 4:**
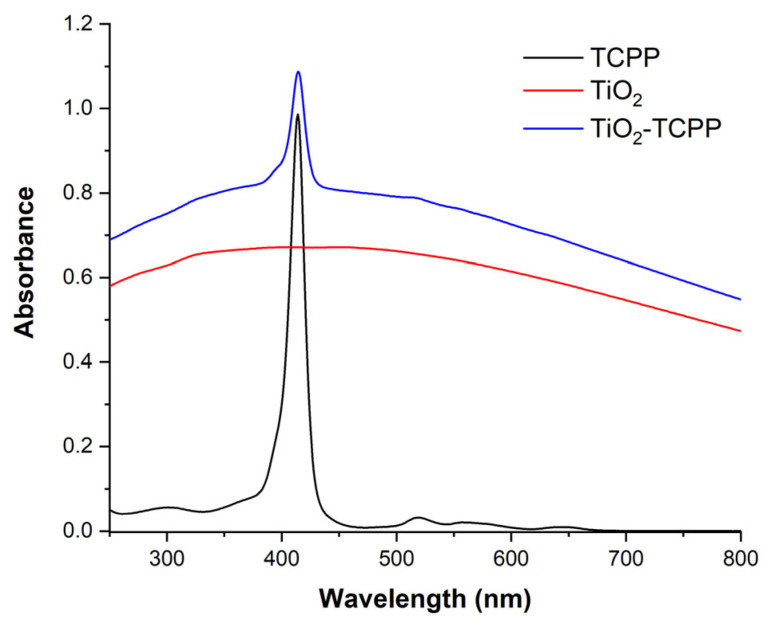
The UV-Vis absorption spectra of TCPP 2.5 μg/mL in PBS, TiO_2_ 50 μg/mL in PBS and TiO_2_-TCPP samples in PBS (diluted eight times).

UV-Vis absorption spectra are shown in Figure 4 for TCPP at 2.5 µg/mL, TiO_2_ at 50 µg/mL, and the TiO_2_-TCPP stock prepared samples diluted eight times. TCPP solution exhibits the characteristic absorption bands for porphyrins: the Soret band at 414 nm and the four Q bands between 500 and 675 nm. However, the Q band II has the shape of a shoulder of Q band III. The appearance of the Soret band at 414 nm shows that there are only TCPP monomers present in this sample. The aspect of the Q bands is influenced by the carboxyphenyl radicals on the porphyrin ring [38]. The Soret band, as well as the Q bands, can also be identified in the spectrum of the TiO_2_-TCPP sample, indicating the presence of TCPP and proving the loading of PS on TiO_2_ NPs.

The quantity of TCPP loaded on NPs, as well as the concentration of TiO_2_ in the TiO_2_-TCPP sample was calculated considering the intensity of the peak at 414 nm and the absorbance value at 700 nm, respectively, as described in [38]. Therefore, TiO_2_-TCPP stock sample contains a concentration of 5.4 μg/mL TCPP and 460 μg/mL of TiO_2_. The functionalization of TiO_2_ NPs with TCPP was previously evidenced through Dynamic Light Scattering (DLS) measurements. It was observed that the hydrodynamic mean size of TiO_2_ was 559.1 ± 126.9 nm, and after loading of PS, the TiO_2_-TCPP complex had a mean size of 1225.8 ± 274.1 nm [38]. Although these dimensions exceed those typically desired for in vivo nanocarriers, the present study focuses on protein–nanocomplexes interaction mechanisms rather than delivery performance. For translational applications, further surface functionalization would be required to reduce aggregation.

**Figure 5:**
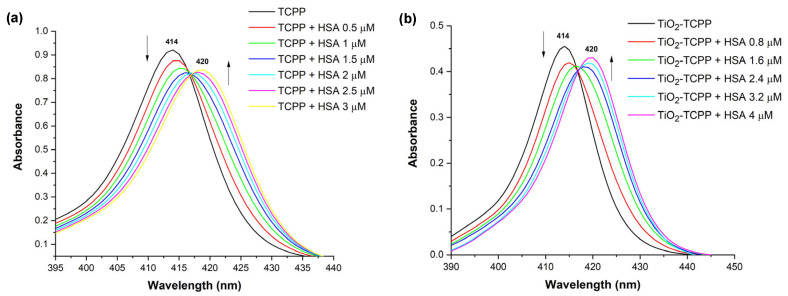
UV-Vis absorption spectra of (**a**) TCPP at a concentration of 3 µM and HSA with concentrations varied between 0 and 3 µM; (**b**) TiO_2_-TCPP (2.7 μg/mL TCPP and 230 μg/mL of TiO_2_) and HSA with concentrations varied between 0 and 4 µM (measured in 0.5 cm cuvettes).

The binding affinity with HSA was determined for TCPP alone and for TiO_2_-TCPP. In both cases, TCPP concentration and TiO_2_-TCPP concentration, respectively, were maintained constant, while HSA concentration was varied. UV-Vis absorption spectra of the samples are shown in Figure 5a,b.

When measuring the interaction of TCPP with HSA, samples with a volume of 1.5 mL of TCPP 6 µM were titrated with 1.5 mL of HSA solutions in PBS, having concentrations between 0 and 6 µM. This way, the concentration of TCPP in all spectra shown in Figure 5a was 3 µM, and the concentration of HSA ([HSA]) varied in the range of 0–3 µM.

For the binding of the TiO_2_-TCPP complex with HSA, 0.5 mL TiO_2_-TCPP stock solutions were titrated with 0.5 mL HSA solutions with a concentration that varied from 0 to 8 µM. TCPP concentration was constant at 2.7 µg/mL (3.4 µM) and TiO_2_ at 230 µg/mL, while the concentration of HSA ranged from 0 to 4 µM, as presented in Figure 5b. To avoid saturation due to the TiO_2_ background, these spectra were measured in 0.5 cm thickness optical cuvettes.

The absorbance plots reveal how the spectral properties are modified in the wavelength range of 390–440 nm, as [HSA] increases. In this wavelength range, HSA does not have absorption features, being present only the Soret band of TCPP. HSA has absorption in the UV region, showing a band with maximum at 277 nm. Therefore, changes in HSA concentration should affect only the absorbance in this region of the spectra. However, Figure 5a clearly shows that adding HSA to TCPP samples causes a decrease in the TCPP absorbance and a shift towards longer wavelengths of the peak corresponding to the Soret band. Furthermore, when increasing the concentration of HSA, the peak continues to shift, with the bathochromic effect being observed at all concentrations. Still, the absorbance of the peak does not follow the same decreasing trend throughout all HSA concentrations. Specifically, samples with [HSA] between 0 and 1.5 μM exhibit a progressive reduction in the absorbance of the Soret band. The sample having [HSA] of 2 μM has a slightly lower absorbance compared to the previous sample, whereas for the next two samples (2.5 μM and 3 μM), an increase in the peak absorbance is observed, and there is a shift up to 420 nm.

Further, UV-Vis absorption spectra of TCPP-loaded TiO_2_ nanoparticles when increasing [HSA] behave in a similar manner, as one may observe in Figure 5b. Namely, the peak at 414 nm simultaneously undergoes a hypochromic effect and a red shift for the concentration range of 0–1.6 μM. The absorbance maximum of the Soret for the sample with [HSA] of 2.4 μM is slightly decreased compared to 1.6 μM. The bathochromic shift continues for the HSA concentration interval 2.4–4 μM, and an increase in the peak absorbance is observed in this concentration range, as previously observed for TCPP binding to HSA.

The formation of complexes between serum albumins and porphyrins or porphyrin-loaded NPs mainly depends on the charge states of porphyrins [39,40]. TCPP, being an anionic porphyrin, is expected to have a strong binding affinity to HSA; this interaction is driven by electrostatic attractions between the negatively charged carboxylate groups of TCPP and the positively charged regions of HSA. UV-Vis absorption spectra changes with HSA concentrations show that both TCCP, as well as TCPP-loaded TiO_2_ NPs bind to HSA, forming complexes.

In order to determine the binding affinity when the complexes are formed, the binding constant (*K_b_*) was calculated using the Benesi–Hildebrand equation as follows [41]:

(1) $$\frac{1}{A_{0}-A}=\frac{1}{K_{b}\left(A_{0}-A_{1}\right)[HSA]}+\frac{1}{A_{0}-A_{1}}$$

where A_0_ is the maximum absorbance value of the Soret band of TCPP or TiO_2_-TCPP respectively, without added HSA; A is the absorbance value of TCPP or TiO_2_-TCPP when increasing concentrations of HSA are added, at the same wavelength as A_0_; and A_1_ represents the value of absorbance for infinite HSA added.

**Figure 6:**
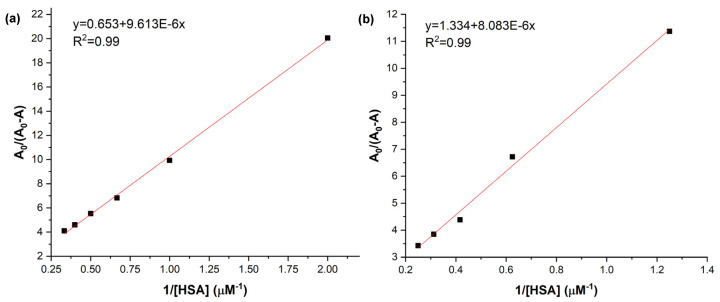
Benesi–Hildebrand plots for interaction of HSA with (**a**) TCPP and (**b**) TiO_2_-TCPP.

Plotting A_0_/(A_0_ − A) vs. 1/[HSA] enables the determination of *K_b_* values from the intercept and slope values for both TCPP alone and TiO_2_-TCPP when interacting with HSA. These plots are presented in Figure 6a,b.

The straight line that fits the data in Figure 6a shows that binding occurs in a singular binding site, implying that one molecule of TCPP binds to one molecule of HSA. Similar, there is a 1:1 binding stoichiometry between TiO_2_-TCPP and HSA (Figure 6b) [32,41].

The binding constant, *K_b_*, describing the interaction strength between TCPP and HSA was calculated from Figure 6a and its value is 1.04 × 10^5^ M^−1^. Figure 6b allowed for the determination of *K_b_* when forming the TiO_2_-TCPP…HSA complex, with the value of 1.23 × 10^5^ M^−1^. The values of *K_b_* determined from UV-Vis absorption data and the one predicted through molecular docking (1.78 × 10^5^ M^−1^) are in good agreement.

It can be noticed that there are no significant changes in the binding affinity with HSA of TiO_2_-TCPP when compare with TCPP alone. That means that the binding properties with HSA for TCPP are not influenced when it is loaded on TiO_2_ nanoparticles.

Several investigations presented analogous modifications of UV-Vis absorption spectra of different porphyrins when interacting with serum albumins, particularly HSA [18,32,39,40]. A similar behaviour to the absorption spectra in Figure 5a was observed in [32], when studying the binding of tetraammonium salt of 5,10,15,20-tetrakis(4′-sulfonatophenyl)-porphyrin (TPPS) with HSA. In this study, the maximum of the Soret band undergoes a bathochromic shift with the increase in [HSA], at the same time suffering a hypochromic effect when [HSA] was between 0 and 0.66 μM, and then there is an increase of the peaks’ absorbance in the range 0.66–2 μM. The results demonstrated that TPPS had a binding affinity towards HSA and molecular docking studies suggested that it is more likely for the binding to take place in close proximity to Sudlow’s site II. A *K_b_* value of 1.41 × 10^6^ M^−1^ was found for the formation of the TPPS…HSA complex [32].

A hypochromic effect of the Soret band of the non-charged 4-TPyP was observed when this was added to the HSA solution, but without the red shift that was recorded in the cases of TPPS and TCPP. This decrease in absorbance was perceived as a proof that 4-TPyP can bind to HSA and slightly alter the structure of albumin. A smaller binding constant of about 10^4^ M^−1^, indicating a moderate interaction was obtained [35].

Another study analyzing the binding of 5,10,15,20-tetrakis(4-N-benzyl-pyridyl)-porphyrin (TBzPyP) to HSA showed a decrease in the peak absorbance, while also moving towards longer wavelengths. In the case of this particular porphyrin, Bordbar et al. [18] suggest an unconventional way of interaction with HSA. Based on the absorption spectra and calculation of the binding constants, it was proposed that the binding happens in two stages. Firstly, one molecule of TBZPyP binds to one molecule of HSA, leading to the formation of a TBZPyP-HSA complex. In the second stage, one molecule of HSA will bind to the newly formed complex. The binding constants calculated based on absorption data were 3.79 × 10^6^ M^−1^ and 1.46 × 10^5^ M^−1^ for the binding of porphyrin derivative TBzPyP with HSA, values that lead to the proposal of a two-stage binding model [18].

A similar behavior of UV-Vis spectra was observed by Pavanelli et al. [39] when adding bovine serum albumin (BSA) to TPPS…CdTe quantum dots functionalized by 3-mercaptopropionic acid (CdTe-3-MPA QD) complex solution. The Soret band undergoes a bathochromic shift from 412 nm to 422 nm and a new band also arises at 436 nm. A binding constant with a value of 3.2 × 10^6^ M^−1^ was calculated for interaction of TPPS_4_ with BSA, at pH 7 [39].

#### 2.2.2. Determination of the Binding Affinity Through LIF Spectroscopy

The association of the TCPP porphyrin and TiO_2_-TCPP nanocomplexes with the HSA protein was also analyzed by LIF. The same concentrations and mixtures as in absorption spectroscopy studies were also employed here.

**Figure 7:**
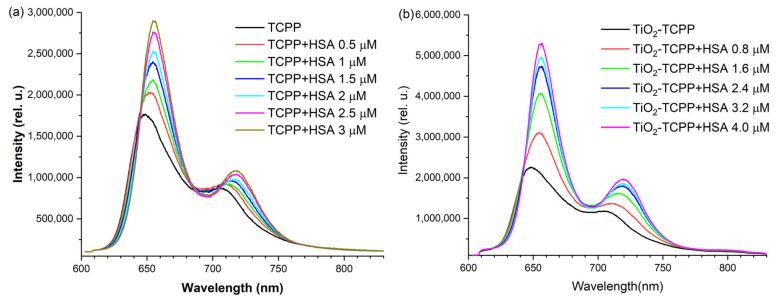
LIF emission spectra for: (**a**) TCPP at a concentration of 3 µM and HSA with concentrations varied between 0 and 3 µM; (**b**) TiO_2_-TCPP (2.7 μg/mL TCPP and 230 μg/mL TiO_2_) and HSA with concentrations varied between 0 and 4 µM.

In Figure 7, the emission fluorescence spectra registered in the range of 600–800 nm, for a 532 nm excitation wavelength, are depicted for titrations of TCPP solutions and TiO_2_-TCPP suspensions at fixed concentrations with varied HSA quantities. As in other systems [42], the binding of porphyrin to HSA or other biologically relevant proteins, induces modifications of fluorescence spectra. Thus, an increase in fluorescence intensity and a red shift of the emission maxima take place for both TCPP and TiO_2_-TCPP solutions.

For TCPP solutions, the fluorescence spectrum shows two emission bands with maxima at 648 and 706 nm. By adding HSA up to 3 µM, a shift of the first band to 655 nm is observed, along with a 1.7-fold increase in intensity. For the emission band placed at a longer wavelength, the red shift is larger, reaching up to 718 nm, but the intensity increase is more modest at about 1.3-fold (Figure 7a). At the excitation wavelength of 532 nm, the absorbance of the samples arising from a Q band of TCPP at a concentration of 3 µM remains very low (0.018–0.020), indicating that the inner filter effect is minimised under these conditions [25].

The red shift of the porphyrin fluorescence maxima with protein addition is in agreement with previous reports for PPIX and HSA complexation and can be an indication that the TCPP is binding within a site of HSA which is less polar than the buffer solution surrounding the protein [19].

For TiO_2_-TCPP complexes, the titration of the suspensions with HSA by varying [HSA] from 0 to 4 µM results in a shift of the first emission band from 648.4 nm to 656.2 nm and a shift of the second band from 707 nm to 720 nm (Figure 7b). While the wavelength displacement behavior is almost similar to that for TCPP bare solutions, the fluorescence intensity enhancement for TiO_2_-TCPP complexes is more pronounced for both peaks with [HSA] increasing, 2.5-fold and 1.8-fold, respectively.

From TCPP ligand fluorescence spectra, when the ligand concentration is kept constant and the protein concentration is varied, information regarding the binding constant and detection limit can be found by plotting the fluorescence intensity function protein concentration taking into account the following formula [43]:

(2) $${(F}_{max}-F_{min})/(F-F_{min})=1+1/{(K}_{b}[HSA])$$

where *F_min_*, *F*, and *F_max_*, are the background-corrected integrated fluorescence areas of the emission bands in the absence of HSA, for HSA concentration [HSA], and for maximum HSA concentration.

**Figure 8:**
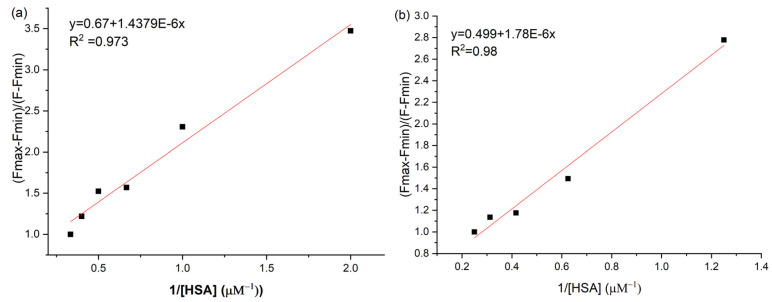
Benesi-Hildebrand plot for determining the binding constant of TCPP with HSA. (**a**) TCPP in PBS at 3µM; the binding constant is 6.9 × 10^5^ M^−1^. (**b**) TiO_2_-TCPP in PBS, 2,7 μg/mL TCPP and 230 μg/mL TiO_2_; the binding constant is 5.6 × 10^5^ M^−1^.

Considering the plots depicted in Figure 8 for TCPP and TiO_2_-TCPP fluorescence variation when HSA is incrementally added to the solutions/suspensions, the binding constants found are 6.9 × 10^5^ M^−1^ and respectively 5.6 × 10^5^ M^−1^. The decrease in the constant value for the nanocomplex compared to the bare porphyrin can be due to the competitive interaction of TiO_2_ with HSA to the detriment of TCPP.

The larger binding constant obtained by fluorescence compared with absorption can be attributed to the higher sensitivity of fluorescence to the microenvironment, aggregation state, and rigidity changes around the porphyrin upon complex formation, whereas absorbance measurements are primarily influenced by macroscopic concentration changes [35].

Porphyrins are known to self-aggregate in aqueous media, and albumin can act as a disaggregating agent [35]. In the present study, the low porphyrin concentration, the absence of significant changes in the background and of the emission spectral profile upon HSA addition suggests that the fluorescence enhancement is not dominated by porphyrin disaggregation, although minor aggregation-related contributions cannot be completely excluded.

Sun et al. [44] showed that the effect of nanoparticles on the fluorescence spectra of HSA is to decrease the fluorescence intensity of protein with a red shift in the emission maximum [45]. The binding of TiO_2_ NPs to HSA involves van der Waals forces, hydrogen bonding, and possibly electrostatic interactions. Synchronous fluorescence spectroscopy indicates that TiO_2_ NPs alter HSA’s conformation, disturbing the environments of tyrosine and tryptophan residues [44]. The binding constant found for TiO_2_ and HSA was of the order of 10^4^ M^−1^, one order of magnitude smaller than the one found by us for the protein and TCPP. This might be the reason for the small decrease in the binding constant to HSA for TiO_2_-TCPP with respect to TCPP.

Other studies on the interaction of serum albumins with porphyrins in nanostructured complexes are scarce in the literature. Parra et al. [40] investigated how bovine serum albumin (BSA) influences the interaction between CdTe quantum dots (CdTe-3-MPA QD) functionalized with 3-mercaptopropionic acid and two water-soluble porphyrins, namely, the positively charged meso-tetra methyl pyridyl porphyrin (TMPyP) and the negatively charged meso-tetrakis(p-sulfonato-phenyl) porphyrin (TPPS). In the case of TMPyP, which has a 4+ charge and forms a charge transfer complex with QD, albumin neither disrupts the existing complex nor reduces its formation probability, likely because the CdTe-3MPA…TMPyP complex formation constant (6.0 × 10^6^ M^−1^) is about one order of magnitude greater than that of TMPyP binding with BSA (7.3 × 10^5^ M^−1^). For TPPS_4_, with a 4− charge, BSA can form a mixed TPPS_4_…QD…BSA complex. TPPS_4_…QD solution shifts the TPPS_4_ absorption band from 412 nm to 422 nm and a new band centered at 436 nm appears. Simultaneously, the TPPS_4_…QD complex fluorescence intensity increases and an emission peak at 650 nm is formed. The binding constant calculated from the fluorescence data is *K*_TPPS_QD + BSA_ = 6.5 × 10^6^ M^−1^ compare to *K*_TPPS_BSA_ = 3.2 × 10^6^ M^−1^. It can be noticed that also in this case, a decrease in the binding constant is observed for the complex with QD when compared with bare porphyrin.

Zhang et al. [46] studied the interaction between a novel porphyrin–dextran coated Fe_3_O_4_ nanoparticle with HSA and found that for the bare porphyrin (5-(4-aminophenyl)-10,15,20-tris-(4-sulfonatophenyl)porphyrin, trisodium salt) the apparent affinity binding constant was 4.98 × 10^4^ M^−1^ compare to 8.56 × 10^3^ M^−1^ for the nanocomplex. The interaction between HSA and the porphyrin was a course of π-π stacking and the steric resistance could be a greater factor. The size of the nanostructure is much bigger than that of the porphyrin; therefore, the *K_A_* value is smaller [46].

#### 2.2.3. Singlet Oxygen Generation

**Figure 9:**
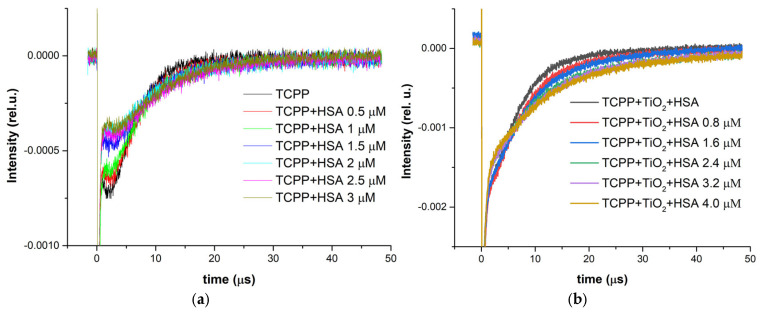
Singlet oxygen phosphorescence kinetics for (**a**) TCPP and (**b**) TiO_2_-TCPP as a function of HSA concentration.

The same samples investigated using LIF excitation at 532 nm were also subjected to time-resolved phosphorescence analysis of generated singlet oxygen. In Figure 9a,b, the ^1^O_2_ phosphorescence signals for TCPP and TiO_2_-TCPP with incremental increases in HSA concentration are presented.

**Figure 10:**
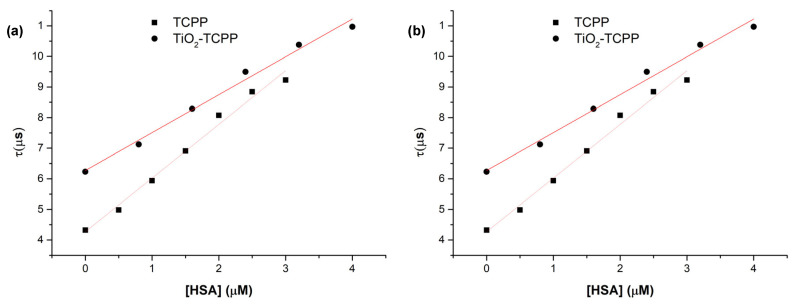
(**a**) The lifetime of singlet oxygen for TCPP and TiO_2_-TCPP as a function of HSA concentration. (**b**) Relative intensity *I/I*o for TCPP and TiO_2_-TCPP as a function of HSA concentration.

From these data, the intensity (*I*) and lifetime *τ* of the phosphorescence emitted by generated singlet oxygen are extracted and plotted in Figure 10. *I*_0_ represents the intensity of the signal when no HSA is added to the samples.

The lifetime of singlet oxygen increases upon the addition of HSA to both TCPP solutions and TiO_2_- TCPP suspensions. In aqueous media, ^1^O_2_ is efficiently quenched by water, resulting in a short lifetime of about 4 μs. The observed lifetime increase can be attributed to the localization of TCPP and the generation of ^1^O_2_ inside or near hydrophobic pockets of has, where water accessibility and, consequently, solvent-mediated quenching are reduced.

It can also be observed that the relative intensity (*I*/*I*_0_) shows a smaller decrease for the TiO_2_–TCPP complex than for free TCPP as the HSA concentration increases. This indicates that singlet oxygen generation is more efficient for the nanocomplex compared with the free porphyrin. Therefore, the binding to HSA has a reduced impact on the photosensitizing properties of TCPP when it is carried by TiO_2_ nanoparticles.

#### 2.2.4. FTIR Spectroscopy

**Figure 11:**
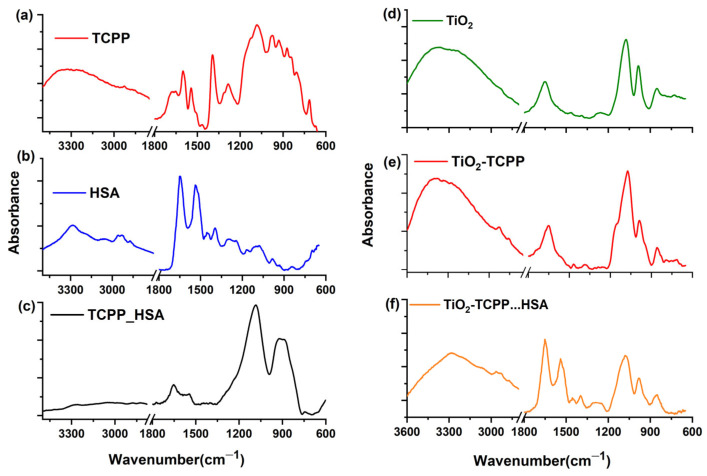
FTIR spectra of: (**a**) TCPP, (**b**) HSA, (**c**) TCPP−HSA, (**d**) TiO_2_ NPs, (**e**) TiO_2_ NPs loaded with TCPP, and (**f**) the TiO2-TCPP…HSA nanocomplex.

The IR spectra recorded in the range of 3300–600 cm^−1^ are shown in Figure 11 for the TCPP porphyrin, HSA protein, and TiO_2_ NP, as well as for their complexes, TCPP−HSA, TiO_2_–TCPP, and TiO_2_–TCPP…HSA.

The full interpretation of the FTIR spectra for TCPP porphyrin, HSA protein, TiO_2_ NPs, and the TiO_2_-TCPP nanocomplex is provided in the Supplementary Materials. More so, the experimental frequencies for TCPP, HSA, and TCPP-HSA complex are centralized in Table S1, and the experimental frequencies for TiO_2_, the TiO_2_-TCPP complex, and the TiO_2_-TCPP…HSA complex are presented in Table S2. Also, the predicted interaction of TiO_2_ with TCPP is presented in the Supplementary Materials.

The binding interactions of TCPP to specific sites of HSA protein were further revealed by analyzing the IR spectrum of TCPP−HSA complex, which is shown in Figure 11c. The broadening and shift of the NH stretching characteristic for the Amide A band for the HSA from 3283 to 3258 cm^−1^ was noted as a consequence of a stabilizing interaction that causes the elongation of the hydrogen bonding. As well, the peaks at 2956 cm^−1^ and at 2872 cm^−1^ (free HSA) specific for the asymmetric/symmetric CH_3_ stretching vibrations were shifted to longer wavenumbers at 2984 cm^−1^ and 2878 cm^−1^ since the complex formation leads to a CH bond contraction and an increase in the respective stretch frequency [47,48]. The disappearance of the maximum at 2930 cm^−1^ specific for the asymmetric CH_2_ vibrational stretching modes was observed after TCPP−HSA interaction. This suggests that significant structural or environmental changes around the CH_2_ groups in HSA may be induced by porphyrin binding. These changes might involve protein conformational shifts, changes in hydration, or direct interactions between porphyrin and HSA that alter the normal vibrational behavior of the methylene groups. This is in good accordance with our molecular docking results and confirm that the contribution of hydrogen bonds plays a crucial role in the formation of the TCPP−HSA complex. Hydrogen bonds optimize the binding site interaction by engaging with AA residues, thereby enhancing the affinity and stability of the complex [49].

The week absorption maximum raised at 1772 cm^−1^ in the FTIR spectrum of TCPP−HSA complex might appear as a shifting result of the TCPP IR absorption peak at 1733 cm^−1^ (due to C=O stretching of the carboxyl groups). This suggests that the carboxyl groups of the porphyrin are involved in the binding process with the protein.

The structure of serum albumins and their interactions with drugs or nanoparticles (NPs) have usually been examined and interpreted by FTIR spectroscopy, mostly through the various amide groups in the peptide chain. Among them, Amide I, arising principally from C=O stretching, is the most extensively studied. The Amide II band results mostly from an out-of-phase combination of NH in-plane bending and CN stretching vibrations. The Amide I and Amide II peaks typically appear in the ranges of 1700–1600 cm^−1^ and 1600–1480 cm^−1^, respectively. Amide bands I and II are useful for the detailed analysis of protein secondary structure, as they are highly sensitive to changes in protein conformation through both the C=O and NH groups, which are involved in the hydrogen bonding formation [50,51,52,53].

For the TCPP-HSA spectrum, a blue spectral shift was observed for the Amide I band from 1647 cm^−1^ in the free HSA to 1654 cm^−1^ for the complex. This may occur due to changes in the protein secondary structures from α-helix to random coil [24,54].

The Amide II band of HSA was also shifted following TCPP binding to protein from 1537 cm^−1^ to 1545 cm^−1^. For similar results Katrahalli et al. suggested that the porphyrin binds with C=O and C−N groups in protein polypeptides. This interaction disrupted the polypeptide’s carbonyl hydrogen bonding network and changed the protein’s α-helix secondary structure [50].

In the spectral range between 1500–1360 cm^−1^ different weak bands specific to TCPP can be observed in the FTIR spectrum of TCPP−HSA complex as result of the CC, C=N, and C=O stretching vibrations, as well as due to CO, OH, and CH porphyrin bending vibrations. These bands slightly change their position in the spectrum (with 5–8 cm^−1^) through lower frequencies. Additionally, the peaks in the Amide III region positioned at 1301 and 1289 cm^−1^ tend to flatten under the influence of the strong absorption maxima appearing at 1084, 921, and 888 cm^−1^, which come from porphyrin moiety and are given by the CC stretching and CH, along with NH bending vibrations. Such results may indicate specific interactions between TCPP and AA residues in HSA.

Frequency changes following the interaction of NP – porphyrin complexes with HSA molecules were detected in the FTIR spectrum of TiO_2_-TCPP…HSA plotted in Figure 11f. The band between 3600 and 3000 cm^−1^ changes its allure as compared to the TiO_2_-TCPP spectrum. The distinctive peak of the NH stretching mode (Amide A) was moved to higher wavenumbers at 3293 cm^−1^. The peaks registered at 2965, 2937, and 2873 cm^−1^ (TiO_2_-TCPP…HSA IR spectrum) appear at higher wavenumbers compared to the TiO_2_-TCPP IR absorption spectrum. This is a consequence of the contraction of the CH bonds coming from the carboxyphenyl radical of the porphyrin coupled with the OH and NH groups of HSA, which causes an increase in their stretching frequency.

Thereby, the locations of the Amide I and Amide II frequencies were monitored to detect changes in the secondary structure of HSA after it is conjugated to the TiO_2_-TCPP nanocomplex. Their characteristic bands also showed a shift to higher wavenumbers, the absorption maxima being recorded at 1651 cm^−1^ and at 1540 cm^−1^. In comparison with HSA interaction with free TCPP (Amide I at 1654 cm^−1^ and Amide II at 1545 cm^−1^), the shift of these bands is smaller in the case of the TiO_2_ nanocomplex. This can be attributed to the fact that the Amide I band of the free HSA (1647 cm^−1^) is influenced by Ti-OH vibration coupled with the C=O and CN stretching of TiO_2_-TCPP (1657 cm^−1^). The Amide II band for the nanocomplex is influenced by the TiO_2_-TCPP band at 1547 cm^−1^ due to CC stretching, CH and NH bending vibrations of the TCPP porphyrin core. These HSA interactions may lead to a reconfiguration of the polypeptide carbonyl hydrogen bonding pattern, which in turn reflects changes in the protein α-helix arrangement [50].

The peak at 1396 cm^−1^ in the TiO_2_-TCPP…HSA spectrum is due to the overlapping of bands specific to individual components in the nanocomplex. Its arising is influenced by the C=O stretch of COO– from the protein [55], the CC, CO, OH, and CH bending from carboxyphenyl radicals, and CC and CN stretching, along with CH bending from the porphyrin ring coupled with the Ti-OH stretching of the NPs [38].

The peaks at 983 and 851 cm^−1^ suffer subtle red shifting from 989 cm^−1^ and 858 cm^−1^ (TiO_2_-TCPP IR spectrum) after the protein linkage to the TiO_2_-TCPP nanocomplex. This can happen due to lengthening of the hydrogen bonding as a consequence of a stabilizing interaction between the chemical components of the nanocomplex [47].

## 3. Materials and Methods

### 3.1. Materials

TCPP was purchased from PorphyChem SAS, Dijon, France, while TiO_2_ NPs as anatase, with 99.95% purity and a 17 nm diameter were supplied by Nanografi (Ankara, Turkey). HSA was purchased from Sigma Aldrich (St. Louis, MO, USA) with a purity of >96%. PBS for solutions and suspensions preparation was provided by Sigma Aldrich (Gillingham, UK).

The TiO_2_-TCPP complexes were synthesised according to the literature protocol [38,56]. A quantity of 4 mg of TiO_2_ powder was added to an 8 mL stock solution of 0.5 mg/mL TCCP in PBS. The suspension was then sonicated for 30 min and after overnight incubation in the dark, it was centrifuged at 10,000 rpm for 10 min. The supernatant was removed, and the precipitate was resuspended in 8 mL of PBS.

To be able to analyze the interaction of TCPP with HSA, stock solutions of TCPP and HSA were prepared in PBS, both having a concentration of 6 µM. Several samples were prepared having varied concentrations of HSA in the range of 0–3 µM and keeping the concentration of TCPP constant (3 µM). For the interaction evaluation of TiO_2_-TCPP with HSA, the same protocol was used, but the concentration of HSA was varied from 0 to 4 µM. The concentration of the HSA stock solution (8 µM) was chosen accordingly to the concentration of TCPP loaded on TiO_2_.

Before measuring the UV-Vis absorption spectra, the samples were kept for 24 h in the dark at room temperature (24 °C).

### 3.2. Molecular Modeling

We have imported the 2D TCPP structure from the PubChem database [57]. The structure was visualized and optimized using our usual protocol for small molecules in MarvinSketch, version 21.20.0 [30,32].

The optimized structure was exported as “.mol2” and converted to “.pdbqt” as required for the molecular docking predictions using Open Babel software, version 3.1.1 [58]. Moreover, using MarvinSketch, we calculate the H-bond acceptor–donor atoms of TCPP.

The 3D structure of HSA was imported from Protein Data Bank (PDB) [59] and optimized by removing water, adding hydrogen atoms, merging the non-polar hydrogen atoms, and adding the Kollman partial charges [59,60,61].

### 3.3. Molecular Docking

Using AutoDock 4.2.6 and following our standard procedure, we predicted the interaction between TCPP and HSA (PDB code of the protein 1AO6 [62]) [63,64]. For our blind molecular docking simulations, we have made a grid box large enough to include the protein. The grid box has the number of specified grid points on x, y, z of 108, 126, 126, a point spacing of 0.542 Å, and the coordinates of the central grid point of maps on x, y, z, are 29.535, 31.826, 23.500.

### 3.4. UV-Vis Absorption Spectroscopy

The absorbance spectra were measured from 200 to 800 nm with 1 nm resolution using a Lambda 950 spectrophotometer (PerkinElmer, Inc., Waltham, MA, USA). The samples were measured in 10 mm-thickness optical quartz cuvettes unless otherwise stated.

### 3.5. Laser Induced Fluorescence Spectroscopy and Singlet Oxygen Measurements

The experimental detection system used to measure laser-induced fluorescence (LIF) spectra and the time-resolved phosphorescence signal of the photosensitized singlet oxygen at a wavelength of 1270 nm was described in detail elsewhere [65,66].

The laser excitation source was the SHG (532 nm) of a Nd:YAG laser (6 ns pulse time width at half maximum, 10 Hz repetition rate, maximum energy per pulse of 25 mJ) (Minilite II, Continuum, Excel Technology, Milpitas, CA, USA).

For LIF measurements, two interconnected optical fibers (M93L02 and M93L01, Thorlabs GmbH, Bergkirchen, Germany, core diameter 1500 µm) were used to collect the fluorescence signal at a 90-degree angle with respect to the incident laser beam. Between the optical fibers, a Notch filter for 532 nm was added to cut the remanent laser radiation from the LIF signal. The emission spectra were analysed by using a SpectraPro SP-2750 spectrograph (Acton Research, Trenton, NJ, USA, Czerny–Turner configuration, 150 lines/mm grating blazed at 500 nm,) coupled with an ICCD camera, a PIMAX 1024RB (Princeton Instruments, Trenton, NJ, USA).

The TCPP molecule is excited to the singlet state and through intersystem crossing forms the triplet state and then transfers the energy to ground state oxygen, generating excited singlet state oxygen (^1^O_2_). The laser spectroscopic technique used for singlet oxygen (^1^O_2_) detection relies on the radiative de-excitation of its excited singlet state, monitored via phosphorescence emission at 1270 nm.

The phosphorescence is detected by a near-infrared (NIR) photomultiplier (PMT Module H10330, Hamamatsu, Japan) and measured with a digital scope (Tektronix DPO-7254, Beaverton, OR, USA). The phosphorescence intensity was obtained by extrapolating the mono-exponential fit of the experimental decay curve to time zero, while the singlet oxygen (^1^O_2_) lifetime was determined from the corresponding decay constant.

### 3.6. FTIR Spectroscopy

FTIR spectroscopy was employed to emphasize the formation of complexes of TCPP and TiO_2_-TCPP with HSA and to assess the interaction through modifications in their characteristic functional groups. Furthermore, FTIR spectra for TCPP porphyrin, HSA protein, TiO_2_ NPs and TiO_2_-TCPP nano complex were analysed in the Supplementary Materials [67,68,69,70,71,72,73,74,75,76,77,78,79,80,81,82,83,84,85,86,87,88,89,90,91,92,93].

Polyethylene (PE) FTIR cards were used to dry samples in quantities ranging from 100 to 300 µL. A larger optical path length was obtained by constantly drying individual 5 µL droplets of material to increase sample thickness. IR spectra were acquired in the 4000–650 cm^−1^ spectral range with a spectral resolution of 4 cm^−1^ using the ATR module of the Thermo Fisher Scientific (Waltham, MA, USA) FTIR Nicolet™ iS™ 50 spectrometer. An average of 16 records was used to obtain each spectrum. The sample was positioned in intimate contact with the ZnSe crystal, which had an internal reflection at a 42° incidence angle, a diameter of 1.5 mm, a refractive index of 2.4, and a penetration depth of 2.03 µm at 1000 cm^−1^.

## 4. Conclusions

This study investigated the interactions between HSA and TCPP, as well as between HSA and the TiO_2_–TCPP complex, by integrating spectroscopic techniques with molecular docking. UV–Vis absorption and laser-induced fluorescence experiments confirmed the formation of both TCPP–HSA and TiO_2_–TCPP…HSA complexes, while FTIR spectroscopy further evidenced the binding sites and revealed the functional groups involved.

Molecular docking predicted that TCPP favorably interacts with amino acid residues located in subdomains IB and IIIA of HSA, suggesting a preferential binding near Sudlow site I. These computational predictions were in agreement with the spectroscopic data. Both free TCPP and TiO_2_ nanoparticles loaded with TCPP were shown experimentally to bind to HSA, and the binding constant determined for TCPP–HSA closely matched the affinity estimated through docking simulations. The conjugation of TCPP with TiO_2_ did not appear to affect the binding constant with HSA as determined by absorption spectroscopy. Due to the higher sensitivity of fluorescence spectroscopy, a slightly lower binding affinity was observed for the TiO_2_–TCPP complex compared with free TCPP. This can be consistent with a competitive contribution from TiO_2_, which partially diminishes the availability of TCPP for interaction with HSA. Detection of generated singlet oxygen showed that binding to HSA has a reduced impact on the photosensitizing properties of TCPP when it is carried by TiO_2_ nanoparticles.

FTIR measurements provided evidence of conformational adjustments in HSA, including modifications of α-helical content and alterations in the hydrogen bonding network within the polypeptide backbone.

Overall, these combined results deepen the understanding of the binding behavior of TCPP and its TiO_2_ nanocomplex with human serum albumin, offering insights into their biological interactions and supporting their potential relevance in photodynamic therapy applications.

## Figures and Tables

**Table 1:** The AAs residues, the interactive forces for the HSA-TCPP complex, and the distance between atoms.

AA Residue	Interactions	Distance (Å)
ARG186	salt bridge	2.68
LYS190	salt bridge	3.15
GLU400	conventional hydrogen bond/Pi–anion	2.35/4.02 or 3.11
ARG428	conventional hydrogen bond/attractive charge	2.57/4.41
LYS432	salt bridge/Pi–cation/Pi–alkyl interactions	2.04/3.66/5.08 or 4.84
LYS436	attractive charge/Pi–alkyl interactions	3.84/4.20
LYS439	conventional hydrogen bond/attractive charge/Pi–alkyl interactions	1.60/4.59/5.35

## Data Availability

The original contributions presented in this study are included in the article/Supplementary Materials. Further inquiries can be directed to the corresponding authors.

## Supplementary Materials

The following supporting information can be downloaded at: https://www.mdpi.com/article/10.3390/ijms27010554/s1.
