# Supplementary material for: Spectroscopic Analysis of the TiO2 Nanoparticles Influence on the Interaction of 5,10,15,20-(Tetra-4-carboxyphenyl)porphyrin with Human Serum Albumin
Source: Int J Mol Sci. 2026 Jan 5;27(1):554. doi: 10.3390/ijms27010554 (PMC12786723; doi:10.3390/ijms27010554)
Supplement: Supplementary file 1 [file ijms-27-00554-s001.zip › ijms-4063531-supplementary.pdf]

Supplementary file

1. Predicted interaction between TiO_2 and TCPP

The interaction between TCPP and TiO2 NP (anatase phase, according to the manufacturer's datasheet) was obtained using SAMSON platform [67] and it is shown in Figure S1. The TiO_2 structure was taken from the Materials Project [68] entry TiO_2 (mp-390), corresponding to anatase with conventional tetragonal lattice parameters a = b = 3.78 Å, c = 9.62 Å, $\alpha = \beta = \gamma = 90.00°$, unit-cell volume 137.57 Å^3, and space group $I4_1/amd$ (No. 141). In SAMSON, the conventional anatase unit cell was replicated 5 × 5 × 5 along the x, y and z directions to generate a larger supercell. The TCPP geometry was taken from the optimized MOL2 structure used in the docking calculations. For the interaction model, we employed the Universal Force Field in SAMSON, without predefined bonds between TiO_2 and TCPP, and used the interactive modeling state updater. The TiO_2–TCPP complex was relaxed by short interactive IM-UFF optimization until the geometry was visually stable.

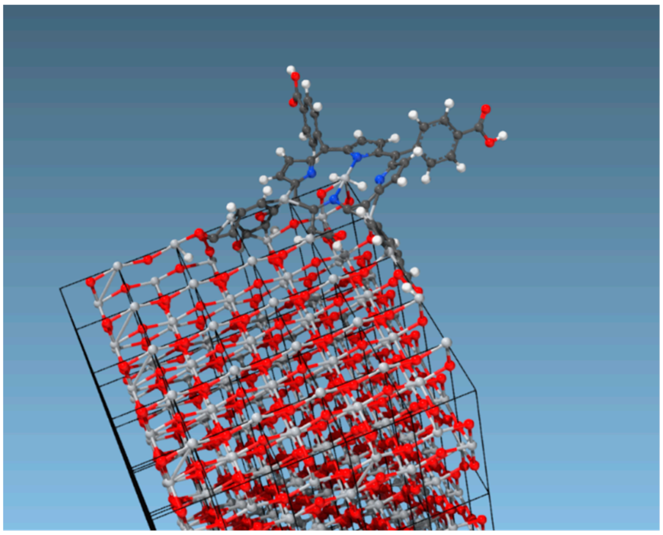

Figure S1. The predicted interactions between TCPP and an anataseTiO_2 NP model.

The simulations indicate that the TiO_2 cluster forms several interactions with TCPP, involving both the carboxyphenyl groups and the nitrogen and carbon atoms of the porphyrin core.

2. FTIR spectroscopy

Table S1 bellow contains the assigned vibrations for maxima in the IR spectrum of TCPP, HSA, and TCPP-HSA complex compounds identifying the chemical bonds responsible for these features.

Table S1. Experimental frequencies for TCPP porphyrin derivative, HSA protein, and TCPP-HSA binding (specific molecular vibrations: ν-stretching, $ν_{as}$ -asymmetric stretching, $ν_s$ symmetric stretching, δ-bending).

TCPP Wave-number (cm^{-1})	Assigned Vibrations	HSA Wave-number (cm^{-1})	Assigned Vibrations	TCPP-HSA Wave-number (cm^{-1})	Assigned Vibrations
**3310**	$ν(CH)_{ring}$, $ν(NH)_{ring}$ ν(OH)	3283	ν(NH)	3258	ν(NH)
**2927**	ν(CH); ν(OH); ν(NH)	2956	$ν_{as}(CH_3)$	2984	$ν_{as}(CH_3)$
**2872**	ν(CH); ν(OH); ν(NH)	2930	$ν_{as}(CH_2)$	2878	$ν_s(CH_3)$
**2854**	ν(CH); ν(OH); ν(NH)	2872	$ν_s(CH_3)$	2824	ν(CH); ν(OH); ν(NH)
**1733**	ν(C=O); δ(OH); ν(CC)			1772	ν(C=O); δ(OH); ν(CC)
**1715**	ν(C=O); δ(OH); ν(CC)				
**1696**	ν(C=O)				
**1683**	ν(C=O)				
**1669**	ν(C=N)				
**1653**	ν(C=N)				
**1647**	ν(CC); δ(CH)	1647	Amide I of proteins ν(C=O)	1654	Amide I of proteins ν(C=O)
**1636**	ν(CC); δ(CH)				
**1603**	$ν(C{=}N)_{ring}$, δ(NH)				
**1540**	ν(CC); δ(CH); δ(NH)	1537	Amide II of proteins δ(N-H) & ν(C-N)	1545	Amide II of proteins δ(N-H) & ν(C-N)
**1507**	ν(CC); δ(CH); $ν(CN)_{ring}$			1500-1360	ν(CC); ν(C=N); ν(C=O); δ(CO); δ(OH); $δ(CH)_{ring}$
**1489**	ν(CC), ν(C=N), ν(C=O)				
**1472**	ν(CC), ν(C=N), ν(C=O)				
**1465**	ν(CC); δ(CH)				
**1457**	ν(CC); δ(CH)	1455	$δ(CH_2)$		

**1393**	δ(CC); δ(CO); δ(OH); δ(CH); ν(CC); ν(CN); δ(CH)ring	1394	ν(C=O) of COO–		
**1339**	δ(CH); δ(OH)	1301	Amide III of proteins (α-helix) ν(C–N)		
**1313**	δ(CH); δ(OH)	1289	Amide III of proteins (β-pleated helix) δ(NH)		
**1284**	ν(CC); δ(CH); δ(OH)	1245	Amide III of proteins (random helix) $\nu_{as}(PO_2)$		
**1081**	ν(CC); δ(CH); δ(NH)	1169	ν_{as}(C–O)	1084	ν(CC); δ(CH); δ(NH)
**1017**	ν(CC); δ(CH), δ(OH)	1081	ν(C–O); $\nu_s(PO_2)$		
**984**	$\nu(CC)_{ring}$; $\nu(CN)_{ring}$; δ(CH)	984	ν_s(PO)		
**968**	$\nu(CC)_{ring}$; $\nu(CN)_{ring}$; δ(CH)				
**930**	δ(CH); δ(NH)	931	ν(C–C) residue	921	ν(C–C); δ(CH); δ(NH)
**872**	δ(CH); δ(NH)			888	δ(CH); δ(NH)
**799**	$\delta(CC)_{ring}$; $\delta(CO)_{ring}$; $\delta(OH)_{ring}$; $\delta(CH)_{ring}$; $\delta(N-H)_{ring}$				
**784**	$\delta(CC)_{ring}$; $\delta(CO)_{ring}$; $\delta(OH)_{ring}$; $\delta(CH)_{ring}$; $\delta(N-H)_{ring}$			741	$\delta(CC)_{ring}$; $\delta(CO)_{ring}$; $\delta(OH)_{ring}$; $\delta(CH)_{ring}$; $\delta(N-H)_{ring}$
**714**	δ(CH); δ(NH); δ(CC)				
**668**	δ(NH), δ(CH), δ(OH), δ(OC=O); δ(CC)			677	δ(OH), δ(OC=O); δ(CC)

In the IR spectrum of TCPP (Figure 8a) the broad band between 3600 – 3200 cm^{-1} is indicative of the O-H stretching vibrations from the carboxylic acid groups, typically broad due to hydrogen bonding [69]. Sharp peaks around 3300 cm^{-1} correspond to the NH stretching vibrations from the pyrrole rings in the porphyrin core. The position may vary slightly depending on the hydrogen bonding and the

overall structure of the porphyrin [70]. The absorption peaks between 3320 – 3070 cm^{-1} are also due to stretching vibrations of CH chemical bonds from porphyrin ring or from carboxyphenyl radicals [38]. The peaks around 2900 cm^{-1} are ascribed to OH/NH groups, as well as to the CH stretching from carboxyphenyl radicals [38,71]. OH bending vibrations give rise to bands in the ranges 1920 – 1720 cm^{-1} and 1370 – 670 cm^{-1}, while CC stretching are responsible for peaks from 1730 to 960 cm^{-1} [38]. C=O stretching vibrations of the carboxyl groups contribute to the formation of absorption bands in the spectral range between 1730 - 1630 cm^{-1} [72]. Several peaks in the region from 1700 to 1600 cm^{-1} are attributed to the typical C=C stretching vibrations in the phenyl rings attached to the porphyrin core [73] and C=N stretching of the porphyrin macrocycle [74]. CH bending vibrations participate in the formation of absorption bands in the range 1690 - 670 cm^{-1}. The absorption bands observed in the IR spectrum of TCPP at 1603 cm^{-1}, 1540 cm^{-1}, 1081 cm^{-1}, and in the range from 870 to 670 cm^{-1} are due to NH and CH bending vibrations [38,75]. Peaks at 1507, 1393, 984, and 968 cm^{-1} are due to the CN stretching vibrations within the porphyrin core [70]. Multiple peaks in the 900 - 700 cm^{-1} spectral range are attributed to CH bending vibrations of the aromatic rings. The bending vibrations of CC chemical bonds contribute to the appearance of peaks between 800 – 670 cm^{-1}. CO bending vibrations, along with other vibrations, are responsible for the appearance of peaks at 799, and 784 cm^{-1}, as well as for the peak at 1393 cm^{-1}. Besides these vibrations, bending vibrations of the OC=O group are responsible for the appearance of the peak observed at 668 cm^{-1} in the IR spectrum of TCPP [38].

The HSA spectrum (Figure 8b) displays numerous well-defined peaks within the 4000 - 600 cm^{-1} spectral window, allowing clearly identification of its specific features. At 3283 cm^{-1} the stretching vibrations of NH bonds can be observed, which gives rise to the amide A band. This band is not sensitive to the conformation of the polypeptide backbone [76]. Asymmetric CH_3 stretching gives rise of the peak at 2956 cm^{-1}, while the asymmetric CH_2 stretching is responsible for the maximum at 2930 cm^{-1}. As well, the peak at 2872 cm^{-1} is influenced by the symmetric CH_3 stretching [77]. For the protein structural studies, the most widely examined modes are amide I, amide II and amide III. Amide I band relates to the α-helix structure of protein [78]. It ranges from 1700 to 1600 cm^{-1} and arises principally from the C=O stretching. Amide II band, ranging from 1600 to 1480 cm^{-1} is resulting mostly from an out-of-phase combination of NH in plane bending and CN stretching vibrations, and is also influenced by the protein backbone structure [79]. The complex vibrations in the Amide III band (1400–1200 cm^{-1}) are mainly triggered by the in-phase combination of NH bending and CN stretching vibrations with small contributions from the CO in plane bending and the CC stretching vibration. Amide III band is influenced by the side chain structure and the backbone of the protein [76]. The Amide I band of HSA in our study is located at 1647 cm^{-1}, and at 1537 cm^{-1} was identified the Amide II of proteins. The peak at 1455 cm^{-1} appears at a consequence of the CH_2 scissoring, and the stretching vibration of C=O bond

(from COO–) give rise to the peak at 1394 cm^{-1}. In the Amide III range, the peak at 1245 cm^{-1} is due to the symmetric PO_2 stretching vibrations. The IR maximum absorption at 1169 cm^{-1} was attributed to the ester CO asymmetric stretch. The CO stretching was also responsible for the appearance of the maximum at 1081 cm^{-1} [77].

Tabel S2 presents the experimental frequencies of the IR bands of TiO_2 NPs, TiO_2 NPs loaded with TCPP porphyrin derivative, and of TiO_2-TCPP…HSA nanostructures, as well as their assigned vibrations.

Table S2. Experimental frequencies for TiO_2, TiO_2-TCPP complex, and TiO_2-TCPP…HSA complex (specific molecular vibrations: ν-stretching, ν_{as} -asymmetric stretching, ν_s symmetric stretching, δ-bending, ω-wagging).

**TiO_2 Wave-number (cm^{-1})**	**Assigned Vibrations**	**TiO_2-TCPP Wave-number (cm^{-1})**	**Assigned Vibrations**	**TiO_2-TCPP… HSA Wave-number (cm^{-1})**	**Assigned Vibrations**
**3381, 3266**	ν(OH)	3378, 3273	ν(OH)	3293	ν(NH)
		2926	ν(CH); ν(OH); ν(NH)	2965, 2937	ν_{as}(CH)
		2858	ν(CH); ν(OH); ν(NH)	2873	ν(CH); ν(OH); ν(NH)
		1740	ν(C=O); δ(OH); ν(CC)		
**1650**	δ(Ti-OH);	1658	δ(Ti-OH); ν(C=O)&ν(C=N)	1651	Amide I of proteins ν(C=O); δ(Ti-OH)
		1647	ν(CC); δ(CH)		
		1580	ν(C=N)$_{ring}$, δ(NH)		
		1564	ν(CC); δ(CH); δ(NH)		
		1547	ν(CC); δ(CH); δ(NH)	1540	Amide II of proteins δ(N-H) & ν(C-N)
		1533	ν(CC); δ(CH); ν(CN)$_{ring}$		
		1517	ν(CC); δ(CH); ν(CN)$_{ring}$		
		1484	ν(CC), ν(C=N), ν(C=O)		
**1466**	δ(CH_2)	1464	δ(CH_2)	1455	δ(CH_2) & ν(CC); δ(CH)

		1407	ν(Ti-O)		
**1391**	ν(Ti-O)	1396	ν(Ti-O)& δ(CC), δ(CO), δ(OH), δ(CH); ν(CC), ν(CN); δ(CH)$_{ring}$	1396	ν(C=O) of COO–& δ(CC), δ(CO), δ(OH), δ(CH); ν(CC), ν(CN); δ(CH)$_{ring}$; ν(Ti-O)
		1364	ν(Ti-O)	1289	Amide III of proteins (β-pleated helix) δ(NH)
				1078	ν(C–O); ν$_s$(PO$_2$)
**985**	ν(Ti–O–Ti); ω(CH)	989	ν(Ti–O–Ti); ω(CH); ν(CC)$_{ring}$; ν(CN)$_{ring}$; δ(CH)	983	ν(CC)$_{ring}$; ν(CN)$_{ring}$; δ(CH); ν$_s$(PO); ν(Ti–O–Ti); ω(CH)
**853**	ν(Ti–O–Ti); ω(CH)	858	ν(Ti–O–Ti); ω(CH)	851	ν(Ti–O–Ti); ω(CH)
**727**	ν(Ti–O)	712	δ(CH); δ(NH); δ(CC)	668	δ(NH), δ(CH), δ(OH), δ(OC=O); δ(CC)

The IR spectrum of the TiO_2 sample (Figure 8d) exhibits peaks assigned to vibrations of physically adsorbed water, surface hydroxyl species, and Ti–O bonds [80], as well as weak influence of precursors used in the nanostructures synthesis. Small crystallites could determine the broadness of the bands [81]. The broad band between 3600 and 3000 cm^{-1} is related to the O–H stretching mode of hydroxyl group, indicating the presence of moisture in the sample [82]. The maximum at 3381 cm^{-1} corresponds to the both symmetric and asymmetric stretching vibrations of the hydroxyl group (Ti–OH) [83]. The shoulder at about 1740 cm^{-1} can be associated to the asymmetric stretching mode of titanium carboxylate coming from the precursors used in the synthesis process [81]. The peak at 1650 cm^{-1} is associated with the O–H bending vibrations of the absorbed water molecules (Ti-OH) [83]. It might be also assigned to deformation vibrations of C-H bonds in the residual isopropoxide groups remaining on the surface of TiO_2 NPs following the synthesis process [80]. Therefore, the two observed peaks at 3381 and 1650 cm^{-1} correspond to the surface adsorbed water and hydroxyl groups [84] and the presence of OH bands in the spectrum was owing to chemically and physically adsorbed H_2O on the surface of nanoparticles [83]. Several weak bands are detectable from 1500 to 1250 cm^{-1} spectral range, which could be due to carboxyl (C═O) and methylene groups from residual organic species left on NPs surface after synthesis [81]. The peak at 1073 cm^{-1} can be also assigned to the surface carbonate adsorbed on TiO_2 NP [85]. These IR absorbances, along with the absorption in the δOH region (in this case at 1650 cm^{-1}) are pH-dependent [86]. The absorption with maximum at 985 cm^{-1} is attributed to surface peroxo species [87]. The IR spectrum of TiO_2 NP shows sharply increasing absorption between 900 to 600 cm^{-1} with peaks at 853

and 727 cm^{-1}, which represent the contribution from the anatase titania. These features are found in the TiO_2 spectral region that is known to contain phonon modes [88,89], and are ascribed to the Ti–O stretching and Ti–O–Ti bridging stretching modes [81].

The IR spectrum of nanocomplex depicted in Figure 8e highlights the structural changes induced by adsorption of TCPP on TiO_2 NPs surface. It presents the absorption bands characteristic to TiO_2 NPs, but also contributions of TCPP IR characteristics. These suggest the loading of porphyrin on the metal oxide nanostructures. The stretching vibrations of CH bonds along with OH and NH stretching can be observed at 2926 and 2858 cm^{-1} in the IR spectrum of TiO_2-TCPP composite, indicating that it contains porphyrin on the surface of TiO_2 NPs. Further observation shows that the alure of band between 3600 and 3000 cm^{-1} characteristics to the OH stretching mode of hydroxyl group is slightly modified indicating changes of TiO_2 surface by adsorption of porphyrin. The peak at 1658 cm^{-1} associated with the Ti–OH bending vibrations is accompanied by the peak at 1647 characteristic for the CC stretching, and CH bending from carboxyphenyl radicals of TCPP molecule. Weak absorption peaks in the spectral range 1565 – 1365 cm^{-1} appear in the IR spectrum of TiO_2-TCPP complex as influence of specific bonds vibrations of porphyrin molecular structure, specifically CC stretching, CH and NH bending, CN stretching from porphyrin ring, CC, CO, OH and CH bending from carboxyphenyl radicals. The attachment of porphyrin to TiO_2 nanostructures is also suggested by the spectral changes observed in the ATR-FTIR spectrum of TiO_2-TCPP from 1200 cm^{-1} to 900 cm^{-1}, where the peak at around 1070 cm^{-1} is preceded by a shoulder with maxima at 1143 and 1108 cm^{-1} coming from CC, CH, and OH bending of porphyrin bonds [38]. The absorption bands between 900 and 600 cm^{-1} might be due to superposition of Ti–O–Ti vibrational modes with NH, CH, CC, CO bending vibrations of TCPP, as well as CC and CN stretching from TCPP porphyrin ring.

An important observation made on the IR spectrum of TiO_2-TCPP is the shifting towards longer wavenumbers (1740 cm^{-1}) of the TCPP characteristic peak at 1733 cm^{-1}, accompanied by the vanishing of some specific bands for TCPP, as example the peaks at 1715 cm^{-1}, 1683 cm^{-1}, 1603 cm^{-1}, and 1314 cm^{-1}. These features correspond to the stretching vibrations of carbonyl groups and the bending vibrations of hydroxyl groups, along with a decrease in the vibrations associated with carboxyphenyl radicals. This suggests that TCPP attaches to TiO_2 through these carboxyphenyl radicals.

Recent reports have shown that TCPP is chemically adsorbed onto the surface of TiO_2, establishing strong Ti–O–C=O linkages. This chemisorption implies a stable and specific interaction between TCPP and TiO_2, which is decisive for their combined applications [90,91]. The changes observed in the IR spectrum of TiO_2-TCPP in our study suggest that the loading of TiO_2 NPs with TCPP may be similar to that described by Kollhoff *et al*. [92], where carboxyl functionalized porphyrins create surface-anchored

connecting carboxylates with TiO_2 NPs through the carboxylic acid radical. Modifications observed in the IR spectrum of the complex obtained by the porphyrin loaded on TiO_2 NPs suggested that the adsorption of porphyrin may involve the pyrroles in the porphyrin ring, like Duan et al. have reported [93], and/or may involve the radicals of the porphyrin derivative, as Kollhoff *et al*. have described in their paper [92]. In another study, the interaction of bovine and human serum albumin with SiO_2 and TiO_2 NPs was investigated [79]. The effect on the secondary structure of proteins examined by FTIR spectroscopy showed that albumin was adsorbed on the surface of nanoparticles in a distinct way. Different changes were discovered in the albumin structure related to the physicochemical properties of each type of particle in the investigation. Shifts in the central frequencies of the Amide I and Amide II bands, as well as modifications in the ratios of the two bands, reflect conformational changes during protein binding to NPs [79].
